# Association of circulating neuregulin 4 with metabolic syndrome in obese adults: a cross-sectional study

**DOI:** 10.1186/s12916-016-0703-6

**Published:** 2016-10-24

**Authors:** Chengfu Cai, Mingzhu Lin, Yanfang Xu, Xuejun Li, Shuyu Yang, Huijie Zhang

**Affiliations:** 1 Department of Otolaryngology Head and Neck Surgery, The First Affiliated Hospital of Xiamen University, Teaching Hospital of Fujian Medical University, Xiamen, China; 2 Department of Endocrinology and Diabetes, The First Affiliated Hospital of Xiamen University, Teaching Hospital of Fujian Medical University, 55 Zhenhai Road, Xiamen, 361003 China; 3 Department of Nephrology, The First Affiliated Hospital, Fujian Medical University, Fuzhou, China; 4 Department of Epidemiology, Tulane University Health Sciences Center, New Orleans, LA USA

**Keywords:** Neuregulin 4, Obesity, Metabolic syndrome, Body fat mass

## Abstract

**Background:**

Neuregulin 4 (Nrg4) is a secreted adipokine recently identified as playing an important role in modulating systemic energy metabolism and the development of obesity-associated disorders. However, information is not available regarding the association between circulating Nrg4 and risk of metabolic syndrome (MetS) in humans.

**Methods:**

We measured serum Nrg4 in 1212 obese adult subjects (aged 40 years or older), with a waist circumference greater than 90 cm for men or 80 cm for women, recruited from the community.

**Results:**

MetS subjects had lower levels of circulating Nrg4 than healthy controls (*P* < 0.01). The prevalence of MetS was higher in subjects with lower levels of circulating Nrg4 compared to those with higher values (67.3 % vs. 57.4 %, *P* < 0.05). Likewise, subjects with low levels of circulating Nrg4 had high prevalence of raised fasting glucose and blood pressure, but there was no association with raised triglycerides and reduced HDL-c. In multivariable logistic regression analyses, increased serum Nrg4 was significantly associated with reduced risk of MetS (OR: 0.603; 95 % CI, 0.439–0.828; *P* = 0.002), adjusting for age, gender, current smoking, alcohol consumption, physical activity, BMI, systolic blood pressure, fasting glucose, triglyceride, HDL-c, HOMA-IR, and body fat mass; however, such associations with serum Nrg4 were not noted for each component of MetS.

**Conclusions:**

These findings indicate that circulating Nrg4 concentrations are inversely associated with risk of MetS in obese Chinese adults, suggesting that circulating Nrg4 concentrations may be a protective factor in the development of MetS.

**Electronic supplementary material:**

The online version of this article (doi:10.1186/s12916-016-0703-6) contains supplementary material, which is available to authorized users.

## Background

Metabolic syndrome (MetS) represents a cluster of multiple interrelated metabolic disorders, including obesity, insulin resistance, dyslipidaemia, hypertension, and hyperglycaemia [1, 2]. MetS has gained importance because of its close association with type 2 diabetes and cardiovascular disease [3, 4]. It has been well documented that excess adiposity is strongly associated with the development of MetS in the general population [5, 6]. Moreover, obesity-associated insulin resistance and low-grade inflammation may largely account for the development of MetS [3, 7]. Previous research has indicated that several cytokines secreted by adipocytes, including resistin, leptin and adiponectin, have pro- and anti-inflammatory properties and are associated with incidence of type 2 diabetes, MetS, and cardiovascular disease [8–10].

Neuregulin 4 (Nrg4) is a secreted adipokine recently identified as playing an important role in modulating systemic energy metabolism and in the development of metabolic disorders in rodent and human obesity, including type 2 diabetes and non-alcoholic fatty liver disease (NAFLD) [11]. As a brown fat-enriched endocrine factor, Nrg4 attenuates hepatic lipogenic signaling and preserves glucose and lipid homeostasis in obesity [12]. However, the relationship between circulating Nrg4 and metabolic disorder in human subjects remains largely unclear. To our knowledge, only one case-control study has tested circulating Nrg4 levels in 87 NAFLD subjects versus 87 non-NAFLD controls, and reported that circulating Nrg4 levels were decreased in NAFLD subjects [13]. Information is not available regarding the association between circulating Nrg4 and risk of MetS in humans. Accordingly, in the current study, we aimed to explore the association between circulating Nrg4 and risk of MetS in obese Chinese adults.

## Methods

### Study participants

Obese adults aged 40 years or older from the Lianqian community, Xiamen, China, were screened with physical examination from April 2011 to December 2013. The details of the study design and methods have been previously reported [14]. A total of 1212 adult obese subjects with a waist circumference greater than 90 cm for men or 80 cm for women were included in the analysis. Of them, 485 participants were randomly selected to further receive magnetic resonance spectroscopy for the measurement of hepatic fat content. All subjects completed a physical examination and a standard questionnaire including social-demographic status, lifestyle habits (i.e. smoking status, alcohol consumption and physical activity using the International Physical Activity Questionnaire), and medical history. Individuals were excluded if they had cancer, current treatment with systemic corticosteroids, biliary obstructive diseases, acute or chronic virus hepatitis, drug-induced liver diseases, total parenteral nutrition, autoimmune hepatitis, Wilson’s disease, or known hyper- or hypothyroidism.

All subjects provided written informed consent. The study protocol was approved by the Institutional Review Board of the First Affiliated Hospital of Xiamen University. The methods were carried out in accordance with the approved guidelines.

### Clinical and biochemical measurements

Body weight was measured by using a spring scale, with participants wearing light clothing without shoes. Height was measured by using a vertical ruler. Body mass index (BMI, weight in kilograms divided by the square of the height in meters) was used as a measure of adiposity. Overweight (including obesity) was defined as BMI of 24 or higher, using the Working Group on Obesity in China criteria [15]. Waist circumference was measured at the level of the umbilicus. Three measurements were obtained with a non-stretchable tape, and the mean value was used for analysis. Blood pressure (BP) was assessed in triplicate using an electronic sphygmomanometer (OMRON Company). The mean values of the three readings were used for analysis. Body fat mass was quantified using the HOLOGIC whole body DXA system (Hologic Inc., Bedford, MA). Intrahepatic triglyceride content was measured by magnetic resonance spectroscopy (^1^H-MRS; Avanto 3.0-T, Siemens AG, Erlangen, Germany) as described previously [16, 17]. Predicted resting energy expenditure was determined using the Harris–Benedict equation [18].

Subjects were instructed to fast for 12 hours before screening. A 75-g oral glucose tolerance tests and blood biochemical measurements were conducted for each subject. Triglycerides, total cholesterol and high-density lipoprotein cholesterol (HDL-c) were measured by enzymatic colorimetric methods with an automatic multichannel chemical analyzer (Hitachi 7450, Tokyo, Japan). Low-density lipoprotein cholesterol (LDL-c) was calculated using Friedewald’s formula. Serum alanine aminotransferase and aspartate aminotransferase were measured by standard enzymatic methods. Serum gamma-glutamyltransferase was measured by the Szasz–Persijn method. Fasting plasma glucose concentrations and 2-h glucose concentrations were measured using the glucose oxidase method. Serum insulin concentrations were measured using an electrochemiluminescence immunoassay (Roche Elecsys Insulin Test, Roche Diagnostics, Mannheim, Germany). Insulin resistance status was assessed using the homeostasis model assessment of insulin resistance (HOMA) according to the following formula: fasting serum insulin (μU/mL) × fasting plasma glucose (mmol/L)/22.5.

### Definition of metabolic syndrome (MetS)

MetS was defined according to International Diabetes Federation diagnostic criteria [19], which included abdominal obesity (waist circumference ≥ 90 cm for Chinese men or ≥ 80 cm for Chinese women), plus two or more of the following: (1) reduced HDL-c (<1.03 mmol/L for men or < 1.29 mmol/L for women, or specific treatment for this lipid abnormality), (2) raised triglyceride level (≥ 1.7 mmol/L, or specific treatment for this lipid abnormality), (3) raised BP (≥ 130/85 mmHg or treatment of previously diagnosed hypertension), or (4) raised fasting plasma glucose (≥ 5.6 mmol/L or previously diagnosed type 2 diabetes).

### Serum Nrg4 measurement

Serum Nrg4 concentrations were measured using an enzyme-linked immunosorbent assay (ELISA) kits (Aviscera Biosciences, Santa Clara, CA). The assay has been shown to be highly sensitive to human Nrg4 with a sensitivity of 0.25 ng/mL. The linear range of the standard was 0.5–32.0 ng/mL, and the intra- and inter-assay variations were both less than 10 %.

### Statistical analysis

Data are presented as means ± standard deviation (SD) or means ± standard error (SEM) or median (interquartile range) for continuous variables or number and percentage for categorical variables. Serum Nrg4, triglycerides and HOMA-IR were log-transformed to improved normality before analytical comparisons. The subjects were classified into two groups according to metabolic status or four quartiles according to serum Nrg4 levels (Quartile 1: < 2.46 ng/mL, Quartile 2: 2.47–3.34 ng/mL, Quartile 3: 3.35–4.73 ng/mL, and Quartile 4: ≥ 4.74 ng/mL).

A χ^2^ test or logistic regression models were used to examine differences in categorical variables in different study groups. Analyses of covariance were performed using general linear models to test differences in study variables between different groups. In addition, we also performed sensitivity analyses stratified by gender or tertiles of serum Nrg4 levels. The correlation of serum Nrg4 levels with metabolic risk factors was analyzed by Pearson correlation coefficients. Multivariable logistic regression models were used to examine the association of serum Nrg4 levels with risks of MetS and components of MetS, adjusted for age, gender, smoking, alcohol consumption, physical activity, BMI, systolic BP, glucose, triglyceride, HDL-c, HOMA-IR, and body fat mass. Two-sided values of *P* < 0.05 were considered statistically significant. All statistical analyses were performed with SAS version 9.3 (SAS Institute, Cary, NC).

## Results

Table 1 summarizes the mean levels of study variables by subtypes of obese subjects. The mean age of the subjects was 53.3 ± 7.3 years. Within the sample, 41.2 % (781/1212) of subjects had MetS. Compared with non-MetS subjects, MetS subjects had an unfavourable metabolic profile, including higher levels of BMI, fasting plasma glucose, postprandial glucose, systolic BP, diastolic BP, triglycerides, total cholesterol, body fat mass, and HOMA-IR, and lower levels of HDL-c. There was no difference in LDL-c between the two groups. Of interest, MetS subjects had lower serum Nrg4 levels than non-MetS subjects (3.24 (2.40–4.52) ng/mL vs. 3.55 (2.60–5.29) ng/mL, *P* < 0.01).

**Table 1:** Clinical characteristics of obese subjects by metabolic syndrome

Variables	Overall	Metabolic syndrome	Non-metabolic syndrome	*P* value
Sample size	1212	781	431	
Age, years	53.3 ± 7.3	54.3 ± 7.2	51.6 ± 7.2	< 0.001
Gender (male), n (%)	347 (28.6)	251 (32.1)	96 (22.3)	< 0.001
BMI, kg/m^2^	27.1 ± 2.7	27.4 ± 2.8	26.6 ± 2.6	< 0.001
Waist circumference, cm	93.8 ± 7.2	94.7 ± 7.4	92.2 ± 6.6	< 0.001
Current smokers, n (%)	158 (13.0)	104 (13.3)	54 (12.5)	0.445
Systolic BP, mmHg	133.3 ± 17.5	139.2 ± 16.4	122.6 ± 14.2	< 0.001
Diastolic BP, mmHg	79.3 ± 10.6	82.3 ± 10.3	73.7 ± 8.7	< 0.001
Triglycerides, mmol/L	1.6 (1.1–2.3)	1.9 (1.4–2.7)	1.5 (1.1–2.0)	< 0.001
Total cholesterol, mmol/L	5.9 ± 1.1	6.0 ± 1.1	5.7 ± 1.0	< 0.001
LDL- cholesterol, mmol/L	3.7 ± 1.0	3.7 ± 1.0	3.6 ± 0.9	0.533
HDL-cholesterol, mmol/L	1.4 ± 0.3	1.3 ± 0.3	1.5 ± 0.3	< 0.001
Fasting glucose, mmol/L	6.1 ± 1.7	6.5 ± 1.9	5.5 ± 0.9	< 0.001
2-h glucose, mmol/L	9.0 ± 4.0	9.9 ± 4.4	7.2 ± 2.3	< 0.001
HOMA-IR	2.92 (2.10–4.25)	2.81 (2.04–4.04)	2.83 (1.84–3.99)	< 0.001
Serum Nrg4, ng/mL	3.35 (2.47–4.74)	3.24 (2.40–4.52)	3.55 (2.60–5.29)	0.002
Body fat mass, kg	24.0 ± 5.3	24.3 ± 5.4	23.5 ± 5.1	0.013

Data are presented as the mean ± SD or median (interquartile range)

*BMI* body mass index, *HOMA-IR* homeostasis model assessment of insulin resistance, *Nrg4* Neuregulin 4

**Fig. 1:**
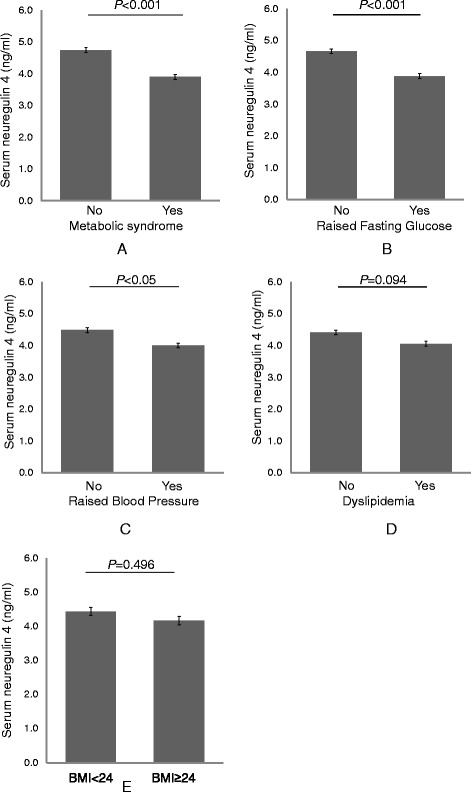
Serum neuregulin 4 (Nrg4) levels according to components of metabolic syndrome (MetS). **a** Serum Nrg4 levels by presence of MetS. **b** Serum Nrg4 levels by presence of raised fasting glucose. **c** Serum Nrg4 levels by presence of raised blood pressure. **d** Serum Nrg4 levels by presence of raised triglycerides or reduced HDL-cholesterol. **e** Serum Nrg4 levels by presence of overweight/obesity; *P < 0.05; **P < 0.01; BMI, body mass index

As shown in Fig. 1, serum Nrg4 levels were significantly reduced in subjects with raised fasting glucose (3.21 (2.38–4.58) vs. 3.53 (2.60–5.05), *P* < 0.001), raised BP (3.24 (2.37–4.59) vs. 3.49 (2.60–5.05), *P* < 0.05), or presence of MetS (3.24 (2.40–4.52) vs. 3.68 (2.62–5.53), *P* < 0.001) compared to their controls, adjusted for age, gender and BMI. However, there was no significant difference in serum Nrg4 levels according to overweight/obesity (BMI ≥ 24) and lipid profiles (including raised triglycerides and reduced HDL-c).

Table 2 presents the clinical characteristic by quartiles of serum Nrg4 levels, adjusted for age and gender. Gender and smoking status were significantly different between quartiles of serum Nrg4 levels. Diastolic BP, total cholesterol, triglyceride, LDL-c, HDL-c, postprandial glucose, and HOMA-IR showed no significant differences among the four quartiles of serum Nrg4 levels, adjusted for age and gender. In addition, resting energy expenditure showed no significant difference among the four groups, adjusted for age and gender. Compared to subjects in the lowest quartile of serum Nrg4 levels, those in the highest quartile had significantly lower levels of BMI, waist circumference, and body fat mass (*P* < 0.05). Fasting glucose was reduced gradually with the increase of serum Nrg4 (*P* < 0.01). Of note, the prevalence of MetS was significantly higher in subjects with lower levels of serum Nrg4 than those with higher values (67.3 % vs. 57.4 %, *P* < 0.05). With respect to individual components of MetS, prevalence of raised fasting glucose and blood pressure was significantly higher in participants with lower levels of serum Nrg4 than those with higher values; however, prevalence of raised triglycerides and reduced HDL-cholesterol showed no significant difference across quartiles of serum Nrg4 levels.

**Table 2:** Clinical characteristics by quartiles of serum neuregulin 4 (Nrg4) levels in obese subjects

Variables	Serum Nrg4 level				*P* value for trend*
	Quartile 1	Quartile 2	Quartile 3	Quartile 4	
Sample size	303	302	304	303	
Serum Nrg4, ng/mL	1.95 (1.56–2.23)	2.88 (2.70–3.10)	3.90 (3.59–4.31)	6.62 (5.41–9.13)	< 0.001
Age, years	52.2 ± 7.7	53.4 ± 7.1	53.4 ± 7.1	54.4 ± 7.1	0.570
Gender (male), n (%)	126 (41.6)	84 (27.8)	77 (25.3)	61 (20.1)	< 0.001
BMI, kg/m^2^	27.5 ± 2.8	27.05 ± 2.7	27.2 ± 2.6	26.7 ± 2.7**	0.104
Resting energy expenditure, kcal/day	1476.6 ± 189.3	1415.4 ± 191.8***	1414.1 ± 165.3***	1384.3 ± 156.0***	0.491
Waist circumference, cm	95.2 ± 7.3	93.4 ± 7.5	93.9 ± 6.9	92.8 ± 7.1**	0.068
Current smokers, n (%)	57 (18.8)	37 (12.3)	35 (11.5)	29 (9.6)	0.005
Systolic BP, mmHg	134.1 ± 17.6	133.0 ± 17.5	134.3 ± 18.3	131.9 ± 16.8	0.286
Diastolic BP, mmHg	79.8 ± 10.3	79.3 ± 10.9	79.6 ± 10.8	78.3 ± 10.5	0.658
Triglycerides, mmol/L	1.6 (1.1–2.3)	1.6 (1.0–2.4)	2.3 (1.1–2.3)	1.5 (1.0–2.2)	0.522
Total cholesterol, mmol/L	5.9 ± 1.1	5.9 ± 1.1	5.9 ± 1.0	5.8 ± 1.1	0.396
LDL-cholesterol, mmol/L	3.7 ± 1.0	3.7 ± 1.0	3.7 ± 0.9	3.6 ± 1.0	0.353
HDL-cholesterol, mmol/L	1.4 ± 0.3	1.4 ± 0.3	1.4 ± 0.3	1.4 ± 0.3	0.302
Fasting glucose, mmol/L	6.3 ± 2.0	6.2 ± 1.9	6.1 ± 1.6	5.9 ± 1.2***	0.009
2-h glucose, mmol/L	9.1 ± 4.3	9.0 ± 4.1	9.0 ± 4.0	8.7 ± 3.6	0.255
HOMA-IR	2.99 (2.09–4.38)	3.08 (2.07–4.26)	2.99 (2.17–4.29)	2.74 (2.05–3.99)	0.229
Body fat mass, kg	24.1 ± 5.4	23.9 ± 5.6	24.4 ± 5.3	23.8 ± 5.1**	0.047
Metabolic syndrome, n (%)	204 (67.3)	205 (67.9)	198 (65.1)	174 (57.4)**	0.006
Components of metabolic syndrome					
Raised blood pressure, n (%)	190 (62.7)	181 (59.7)	175 (57.6)	162 (53.3)**	0.021
Raised fasting glucose, n (%)	191 (63.0)	188 (61.8)	174 (57.2)**	164 (54.0)***	< 0.001
Raised triglycerides, n (%)	141 (46.5)	143 (47.2)	144 (47.4)	121 (39.9)	0.446
Reduced HDL-cholesterol, n (%)	76 (25.1)	113 (37.3)***	95 (31.3)	98 (32.3)	0.521

Data are presented as the mean ± SD or median (interquartile range)

*Adjusted for age and gender

**P < 0.05 compared with Q1 of serum Nrg4

***P < 0.01 compared with Q1 of serum Nrg4

*Nrg4* Neuregulin 4, *BMI* body mass index, *BP* blood pressure, *HOMA-IR* homeostasis model assessment of insulin resistance

Clinical characteristic by gender and tertiles of serum Nrg4 levels was substantially similar to those shown in Table 2 (Additional file 1: Table S1). Male subjects had lower levels of serum Nrg4 than female subjects, adjusted for age, current smoking, alcohol consumption and physical activity (3.00 (2.12–4.01) ng/mL vs. 3.52 (2.62–5.07) ng/mL, *P* < 0.01). Compared to female subjects in the first tertile of serum Nrg4 levels, those in the third tertile had significantly lower levels of systolic BP, waist circumference, fasting glucose and postprandial glucose (*P* < 0.05). Both male and female subjects with lower levels of serum Nrg4 had higher prevalence of MetS than those with higher values (both *P* < 0.05). Additionally, there was no significant difference in serum Nrg4 levels according to smoking status (Additional file 1: Table S2).

Intrahepatic triglyceride content and liver enzymes were assessed by quartiles of serum Nrg4 levels in 485 obese adults (Additional file 2: Figure S1). Intrahepatic triglyceride content was reduced gradually with the increase in serum Nrg4 levels (15.5 ± 10.8 % in quartile 1, 14.9 ± 10.7 % in quartile 2, 12.9 ± 9.2 % in quartile 3, 12.0 ± 9.0 % in quartile 4, *P* < 0.05 for trend). Serum alanine aminotransferase, aspartate aminotransferase and gamma-glutamyltransferase showed no significant differences among the four quartiles of serum Nrg4 levels (all *P* > 0.05).

As shown in Table 3, serum Nrg4 levels were significantly correlated with BMI, waist circumference, LDL-c, fasting glucose and body fat mass, adjusting for age, gender, smoking, alcohol consumption and physical activity.

**Table 3:** Clinical correlates of serum neuregulin 4 (Nrg4) levels with metabolic risk factors

	Correlation coefficient	*P* value	Multiple adjusted *P* value*
Age, years	0.105	< 0.001	–
Gender (male = 1, female = 2)	–	< 0.001	–
BMI, kg/m^2^	–0.099	< 0.001	0.011
Waist circumference, cm	–0.112	< 0.001	0.034
Systolic BP, mmHg	–0.038	0.190	0.381
Diastolic BP, mmHg	–0.047	0.105	0.944
Total cholesterol, mmol/L	–0.019	0.512	0.079
Total triglycerides, mmol/L	–0.053	0.067	0.617
LDL-cholesterol, mmol/L	–0.015	0.602	0.025
HDL-cholesterol, mmol/L	0.049	0.091	0.464
Fasting glucose, mmol/L	–0.098	< 0.001	0.007
HOMA-IR	–0.047	0.104	0.266
Body fat mass, kg	–0.0005	0.988	0.013

*BMI* body mass index, *BP* blood pressure, *HOMA-IR* homeostasis model assessment of insulin resistance

*Adjusted for age, gender, smoking, alcohol consumption, and physical activity

The multivariable-adjusted odds ratios (ORs) for the association between serum Nrg4 levels and components of MetS are shown in Table 4. After adjustment for age, gender, current smoking, alcohol consumption and physical activity, increased serum Nrg4 was significantly associated with reduced risk of raised fasting glucose and prevalence of MetS (OR: 0.809; 95 % CI, 0.657–0.997 and 0.769; 0.621–0.952, respectively); however, serum Nrg4 was not significantly associated with risks of raised blood pressure (*P* = 0.057), raised triglycerides (*P* = 0.899) or reduced HDL-cholesterol (*P* = 0.991). Furthermore, increased serum Nrg4 was significantly associated with reduced risk of MetS (OR: 0.615; 95 % CI, 0.450–0.841; *P* = 0.002), even after adjusting for age, gender, current smoking, alcohol consumption, physical activity, BMI, systolic BP, fasting glucose, triglycerides and HDL-c; however, such associations of serum Nrg4 were not noted for each component of MetS. After further adjustment for HOMA-IR and body fat mass, the relationship between serum Nrg4 and MetS remained significant (OR: 0.603; 95 % CI, 0.439–0.828; *P* = 0.002).

**Table 4:** Odds ratios of components of metabolic syndrome according to serum neuregulin 4 (Nrg4)

Components of metabolic syndrome	OR	95 % CI	*P* value
Model 1			
Raised blood pressure	0.814	0.658–1.006	0.057
Raised fasting glucose	0.809	0.657–0.997	0.047
Raised triglycerides	0.987	0.806–1.208	0.899
Reduced HDL-cholesterol	0.999	0.803–1.243	0.991
Metabolic syndrome	0.769	0.621–0.952	0.016
Model 2			
Raised blood pressure	0.824	0.662–1.026	0.084
Raised fasting glucose	0.838	0.674–1.042	0.112
Raised triglycerides	1.034	0.838–1.277	0.754
Reduced HDL-cholesterol	1.047	0.836–1.312	0.689
Metabolic syndrome	0.615	0.450–0.841	0.002
Model 3			
Raised blood pressure	0.822	0.660–1.024	0.080
Raised fasting glucose	0.806	0.635–1.022	0.075
Raised triglycerides	1.023	0.827–1.266	0.834
Reduced HDL-cholesterol	1.048	0.837–1.314	0.681
Metabolic syndrome	0.603	0.439–0.828	0.002

*OR* odds ratio, *CI* confidence interval, *BMI* body mass index, *HOMA-IR* homeostasis model assessment of insulin resistance

Model 1: adjusted for age, gender, smoking, alcohol consumption and physical activity

Model 2: adjusted for model 1 + BMI, SBP, glucose, total cholesterol, triglyceride and HDL-c

Model 3: adjusted for model 2 + HOMA-IR and body fat mass

## Discussion

Nrg4 is a brown fat-enriched endocrine factor recently been shown to improve obesity-associated disorders, including type 2 diabetes and NAFLD [12, 20]. In the present study, we provide, for the first time, evidence that circulating Nrg4 concentrations are significantly reduced in subjects with MetS as well as those with particular components. Furthermore, lower circulating Nrg4 concentrations were independently associated with increased risk of MetS in obese Chinese adults. These findings indicate that circulating Nrg4 could be a protective factor in the pathogenesis of MetS.

Brown adipose tissue activates uncoupled respiration in response to cold temperature and contributes to systemic metabolic homeostasis [21–23]. It has been established that brown adipose tissue is metabolically active in adult humans and correlated with body composition and energy metabolism [24–26]. Activation of brown adipose tissue increases energy expenditure and leads to reduced adiposity, which has provided new insight into the prevention of obesity and MetS [11, 27, 28]. Wang et al. [12] reported that the brown fat-enriched secreted factor Nrg4 attenuated hepatic lipogenic signaling and preserved glucose and lipid homeostasis in obesity. Unfortunately, information regarding the association of circulating Nrg4 with hepatic fat content and energy expenditure in humans is limited. Our data demonstrated that circulating Nrg4 was not significantly associated with predicted resting energy expenditure, suggesting that Nrg4 may not directly engage in brown fat thermogenesis [12]. A case-control study also reported that circulating Nrg4 was significantly decreased in 87 NAFLD subjects versus 87 non-NAFLD controls [13]. In the present study, we provided new clinical evidence revealing that serum Nrg4 levels were inversely associated with hepatic fat content in obese adults (Additional file 2: Figure S1). These data suggest that Nrg4 appears to be involved in crosstalk between brown fat tissue and obesity-associated disorders, including NAFLD and MetS. However, the potential roles of circulating Nrg4 in contributing to obesity-associated disorders and the associations of circulating Nrg4 with metabolic risk factors and MetS remain to be elucidated in population-based studies.

Previous studies have indicated that adipose tissue Nrg4 expression was reduced in obesity and negatively correlated with body fat mass in humans [12, 29], but the association of circulating Nrg4 with body fat mass has been not yet studied. Our findings indicate that circulating Nrg4 is negatively correlated with body fat mass in obese adults. In contrast, Dai et al. [13] reported that circulating Nrg4 was not significantly associated with BMI in a case-control study of 87 NAFLD subjects versus 87 non-NAFLD controls. In the present study, with a sample size of more than 1000 obese adults, we found that circulating Nrg4 was negatively associated with waist circumference and BMI. Therefore, these data indicate that Nrg4 insufficiency may be a common feature of obesity in the general population.

Excess adiposity is associated with MetS as well as metabolic risk factors, i.e. hyperglycaemia, elevated BP and dyslipidaemia [8, 30, 31]. Nrg4 has been identified as being involved in crosstalk between brown fat tissue and obesity-associated disorders [32]. However, limited evidence is available regarding whether circulating Nrg4 is involved in the development of MetS in adults. Dai et al. [13] did not find a significant association of circulating Nrg4 with fasting glucose in 174 NAFLD and non-NAFLD subjects. Kang et al. [33] reported that circulating Nrg4 levels were significantly higher in 57 patients with diabetes mellitus compared with 59 controls without diabetes and were correlated with the serum glucose level and insulin resistance. It should be noted that limited evidence is based on case-control study designs, and sample sizes of all of the studies above are quite small (less than 200 subjects). In contrast, our data demonstrate that circulating Nrg4 is significantly decreased in individuals with hyperglycaemia compared to their controls and inversely associated with fasting glucose in more than 1000 obese adults. These findings suggest that low circulating Nrg4 is associated with increased risk of diabetes. Prospective cohort studies are needed to confirm this finding and elucidate the potential underlying mechanisms. In addition, our data indicates that circulating Nrg4 is significantly decreased in individuals with elevated blood pressure, whereas there is no significant difference in circulating Nrg4 levels in individuals with raised triglycerides or reduced HDL-c compared to controls.

Interestingly, our data demonstrates that circulating Nrg4 was significantly decreased in individuals with MetS compared to controls and independently associated with risk of MetS. To date, this is the first population-based study to evaluate the association of circulating Nrg4 and MetS. The findings indicate that each SD increase in circulating Nrg4 levels (log-transformed) is associated with a 38.5 % decrease in the risk of MetS. Nrg4 is expressed in adipocytes, the deficiency of which exacerbates diet-induced insulin resistance and obesity in animal models [12, 29]. However, adjustment for insulin resistance and body fat mass did not attenuate the association of circulating Nrg4 with risk of MetS in the current study. These findings indicate that low circulating Nrg4 appears to add to the risk of MetS independently of obesity and insulin resistance. Indeed, our findings show that circulating Nrg4 is significantly associated with fatty liver, which may play a role in the development of MetS [34]. However, the effect of Nrg4 in the development of MetS needs to be further studied in vivo and in vitro. As a novel endocrine factor, circulating Nrg4 may activate the receptor kinases ErbB3 and ErbB4 and coordinate glucose and lipid homeostasis in obesity [12, 35]. Therefore, Nrg4 may play a potential role in therapeutic developments for MetS.

This community-based, cross-sectional study provided an opportunity to determine the role of circulating Nrg4 in predicting the development of MetS. Nevertheless, there are several limitations to the current study. First, the study was based on cross-sectional data with a relatively limited sample size, and the population consisted of only obese adults. Therefore, further studies are warranted to determine the role of circulating Nrg4 in the development of MetS in the general population. Second, given its cross-sectional design, it is not possible to determine a causal relationship between circulating Nrg4 and the development of MetS. Therefore, the causal association between circulating Nrg4 and MetS should be further evaluated in prospective cohort studies with larger sample sizes and long follow-up periods.

## Conclusions

Our study provides, for the first time, clinical evidence revealing that circulating Nrg4 concentrations are inversely associated with risk of MetS in obese Chinese adults. These findings suggests that circulating Nrg4 concentrations may be a protective factor in the development of MetS and underscore the importance of Nrg4 as a potential therapeutic target for MetS.

## Additional files

Additional file 1: Table S1. Clinical characteristics by gender and tertiles of serum neuregulin 4 (Nrg4) levels. **Table S2.** Serum Nrg4 levels according to smoking status. (DOCX 27 kb)
Additional file 2: Figure S1. Intrahepatic triglyceride content and liver enzymes by quartiles of serum neuregulin 4 (Nrg4) levels in 485 obese adults. (PPTX 70 kb)
