# Supplementary material for: Association of circulating neuregulin 4 with metabolic syndrome in obese adults: a cross-sectional study
Source: BMC Med. 2016 Oct 24;14:165. doi: 10.1186/s12916-016-0703-6 (PMC5075753; doi:10.1186/s12916-016-0703-6)
Supplement: Additional file 1: Table S1. — Clinical characteristics by gender and tertiles of serum neuregulin 4 (Nrg4) levels. Table S2. Serum Nrg4 levels according to smoking status. (DOCX 27 kb) [file 12916_2016_703_MOESM1_ESM.docx]

Table S1.Clinical characteristics by gender and tertiles of serum neuregulin 4 (Nrg4) levels

| Variables | Male | | | |  | Female | | | | P-value ^b^ |
| --- | --- | --- | --- | --- | --- | --- | --- | --- | --- | --- |
|  | Tertile 1 | Tertile 2 | Tertile 3 | P-value^a^ |  | Tertile 1 | Tertile 2 | Tertile 3 | P-value^a^ |  |
| Sample size | 115 | 116 | 116 |  |  | 288 | 289 | 287 |  |  |
| Serum Nrg4 (ng/ml) | 1.77(1.32-2.11) | 3.00(2.69-3.22) | 4.81(4.01-7.13) | <0.001 |  | 2.30(1.92-2.62) | 3.52(3.19-3.90) | 6.10(5.07-8.24) | <0.001 | <0.001 |
| Age (years) | 52.3±7.8 | 52.5±8.0 | 54.9±6.9 | 0.017 |  | 52.8±7.2 | 53.4±7.0 | 53.9±7.2 | 0.164 | 0.748 |
| BMI (kg/m^2^) | 27.9±2.5 | 27.9±2.9 | 27.95±2.6 | 0.676 |  | 27.0±2.8 | 26.9±2.7 | 26.5±2.6 | 0.114 | <0.001 |
| Resting energy expenditure, kcal/day | 1648.9±143.3 | 1644.9±187.1 | 1614.3±134.8 | 0.186 |  | 1345.1±101.5 | 1336.1±98.1 | 1329.4±91.4 | 0.150 | <0.001 |
| Waist circumference (cm) | 98.0±5.8 | 97.7±7.3 | 97.6±5.5 | 0.941 |  | 92.8±7.5 | 92.4±6.8 | 91.5±6.6† | 0.033 | <0.001 |
| Current smokers (n, %) | 50(43.5) | 53(45.3) | 47(40.5) | 0.759 |  | 1(0.4) | 2(0.7) | 5(1.7) | 0.194 | <0.001 |
| Systolic BP (mmHg) | 139.1±15.2 | 137.7±16.7 | 137.0±15.9 | 0.324 |  | 131.1±17.3 | 133.4±19.0 | 129.9±16.9 | 0.035 | <0.001 |
| Diastolic BP (mmHg) | 83.4±9.3 | 83.1±10.8 | 82.3±10.1 | 0.759 |  | 77.7±10.5 | 78.7±10.7 | 77.0±10.1 | 0.132 | <0.001 |
| Triglycerides (mmol/L) | 2.0(1.4-2.6) | 1.9(1.2-2.8) | 1.9(1.3-2.8) | 0.695 |  | 1.5 (1.0-2.1) | 1.5 (0.99-2.1) | 1.4 (1.0-2.1) | 0.734 | <0.001 |
| Total cholesterol (mmol/L) | 6.0±1.1 | 5.8±1.0 | 5.8±1.0 | 0.214 |  | 6.0±1.2 | 5.9±1.0 | 5.8±1.1 | 0.121 | 0.638 |
| LDL- cholesterol (mmol/L) | 3.7±1.1 | 3.5±1.0† | 3.6±1.0 | 0.119 |  | 3.7±1.0 | 3.7±0.9 | 3.6±1.0† | 0.079 | 0.240 |
| HDL-cholesterol(mmol/L) | 1.2±0.3 | 1.2±0.2 | 1.2±0.2 | 0.292 |  | 1.4±0.3 | 1.4±0.3 | 1.4±0.3 | 0.215 | <0.001 |
| Fasting glucose (mmol/L) | 6.4±1.8 | 6.1±1.3 | 6.3±2.2 | 0.361 |  | 6.4±2.3 | 6.0±1.3‡ | 6.1±1.6‡ | <0.001 | 0.042 |
| 2-h glucose (mmol/L) | 9.2±4.4 | 8.5±3.5 | 9.4±5.0 | 0.361 |  | 9.4±4.5 | 8.7±3.6† | 8.7±3.3† | 0.014 | 0.813 |
| HOMA-IR | 2.82(1.93-4.24) | 3.18(2.19-4.34) | 2.70(2.06-4.43) | 0.211 |  | 3.09(2.16-4.42) | 3.03(2.09-4.18) | 2.83(2.07-3.99) | 0.212 | 0.680 |
| Body fat mass (kg) | 21.4±4.2 | 21.4±5.4 | 20.7±4.2 | 0.718 |  | 25.6±5.3 | 25.4±5.2 | 24.7±4.9 | 0.116 | <0.001 |
| Metabolic syndrome (n, %) | 92(80.0) | 85(73.3) | 74(63.8) | 0.022 |  | 185(64.2) | 187(64.7) | 158(54.9) | 0.024 | <0.001 |
| Components of metabolic syndrome | |  |  |  |  |  |  |  |  |  |
| Raised blood pressure (n, %) | 92(80.0) | 78(67.2) | 80(69.0) | 0.064 |  | 161(55.7) | 161(55.7) | 136(47.1) | 0.055 | <0.001 |
| Raised fasting glucose (n, %) | 79(68.7) | 75(64.1) | 53(45.7) | <0.001 |  | 183(63.3) | 167(57.8) | 160(55.4) | 0.137 | 0.833 |
| Raised triglycerides (n, %) | 68(59.1) | 69(59.0) | 68(58.6) | 0.997 |  | 116(40.3) | 119(41.2) | 109(37.9) | 0.700 | <0.001 |
| Reduced HDL-cholesterol (n, %) | 20(17.4) | 29(24.8) | 26(22.4) | 0.377 |  | 102(35.4) | 108(37.4) | 97(33.7) | 0.651 | <0.001 |

Nrg4=Neuregulin 4; BMI=body mass index; HOMA-IR=homeostasis model assessment of insulin resistance.

Data are presented as the mean±SD or median (interquartile range).

^a^, P-values were adjusted for age.

^b^, P-values for gender difference were adjusted for age.

† P< 0.05 compared with Tertlie1 of serum Nrg4 in male and female subjects, respectively.

‡ P< 0.01 compared with Tertile 1 of serum Nrg4 in male and female subjects, respectively.

Table S2.Serum neuregulin 4 (Nrg4) levels according to smoking status.

|  | Serum Nrg4 (ng/ml) | | | P-value for trend§ |
| --- | --- | --- | --- | --- |
|  | Sample size(Smoker/Total) | Smoker | Non-smoker |  |
| All participants | 158/1212 | 2.95(2.13-3.98) | 3.41(2.55-4.83) | 0.623 |
| MetS | 104/781 | 2.79(2.10-3.88) | 3.29(2.52-4.62) | 0.513 |
| Non-MetS | 54/431 | 3.17(2.42-4.00) | 3.65(2.62-5.39) | 0.695 |
| Male | 150/347 | 2.87(2.12-3.86) | 3.05(2.10-4.18) | 0.915 |
| Female | 8/865 | 6.12(3.64-8.47) | 3.52(2.62-5.05) | 0.149 |

Nrg4=Neuregulin 4; MetS=metabolic syndrome; Data are presented as the median (interquartile range).

§: adjusted for age and gender.
