# Supplementary material for: Association of circulating neuregulin 4 with metabolic syndrome in obese adults: a cross-sectional study
Source: BMC Med. 2016 Oct 24;14:165. doi: 10.1186/s12916-016-0703-6 (PMC5075753; doi:10.1186/s12916-016-0703-6)
Supplement: Additional file 2: Figure S1. — Intrahepatic triglyceride content and liver enzymes by quartiles of serum neuregulin 4 (Nrg4) levels in 485 obese adults. (PPTX 70 kb) [file 12916_2016_703_MOESM2_ESM.pptx]

## Slide 1
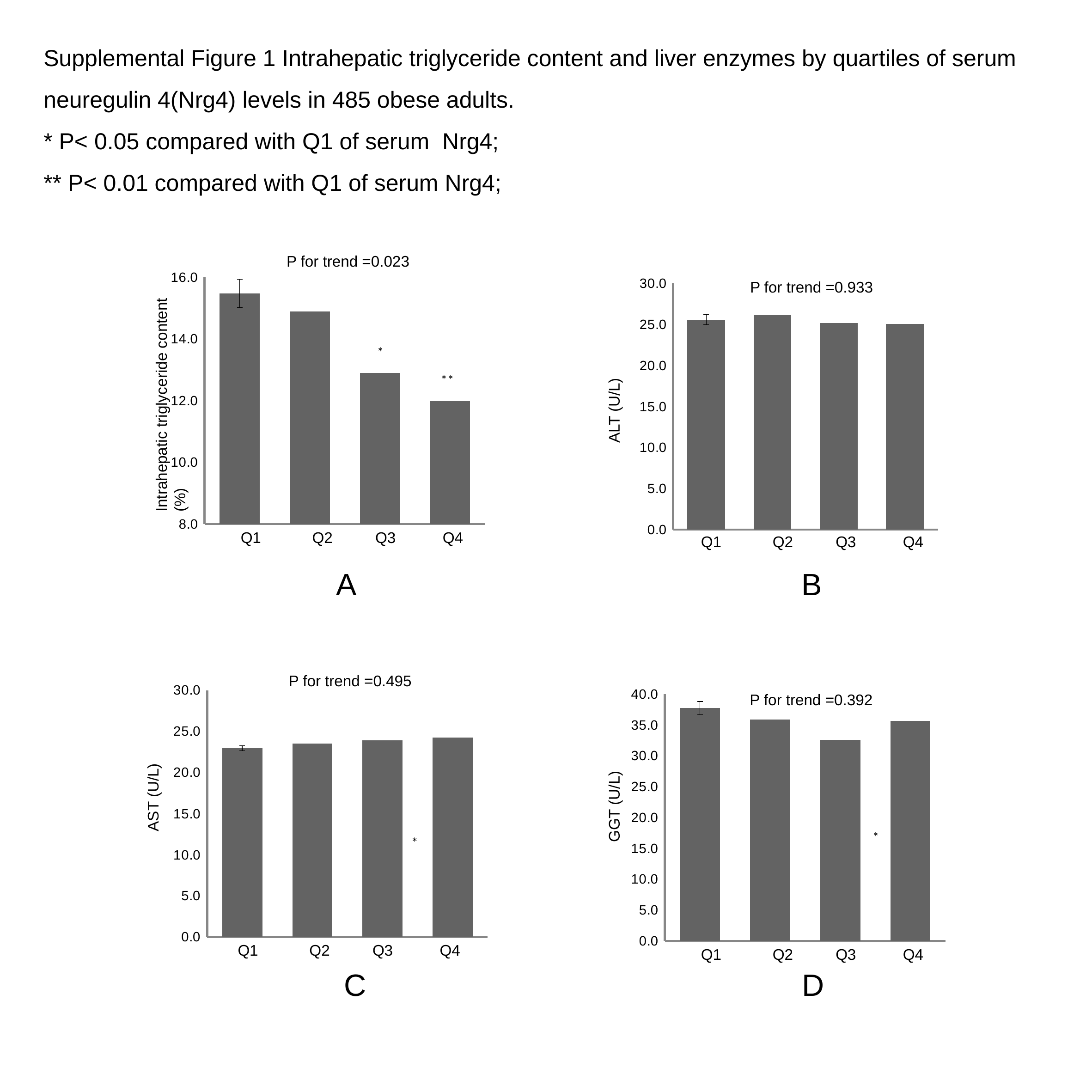

Supplemental Figure 1 Intrahepatic triglyceride content and liver enzymes by quartiles of serum neuregulin 4(Nrg4) levels in 485 obese adults.
* P< 0.05 compared with Q1 of serum Nrg4;
** P< 0.01 compared with Q1 of serum Nrg4;
P for trend =0.023
Intrahepatic triglyceride content (%)
**
Q1 Q2 Q3 Q4
### Chart
| Category | |
|---|---|*
### Chart
| Category | |
|---|---|P for trend =0.933
ALT (U/L)
Q1 Q2 Q3 Q4
A
B
P for trend =0.495
AST (U/L)
*
Q1 Q2 Q3 Q4
### Chart
| Category | |
|---|---|
### Chart
| Category | |
|---|---|P for trend =0.392
GGT (U/L)
*
Q1 Q2 Q3 Q4
C
D
